# 3D printing of mineral–polymer bone substitutes based on sodium alginate and calcium phosphate

**DOI:** 10.3762/bjnano.7.172

**Published:** 2016-11-21

**Authors:** Aleksey A Egorov, Alexander Yu Fedotov, Anton V Mironov, Vladimir S Komlev, Vladimir K Popov, Yury V Zobkov

**Affiliations:** 1 A. A. Baikov Institute of Metallurgy and Materials Science, Russian Academy of Sciences, Leninsky prospect 49, 119334, Moscow, Russia; 2 Institute of Photonic Technologies, Federal Scientific Research Centre "Crystallography and Photonics", Russian Academy of Sciences, 2 Pionerskaya St., 142092 Troitsk, Moscow, Russia

**Keywords:** 3D printing, bone graft, calcium phosphate, composite materials, sodium alginate, tissue engineering

## Abstract

We demonstrate a relatively simple route for three-dimensional (3D) printing of complex-shaped biocompatible structures based on sodium alginate and calcium phosphate (CP) for bone tissue engineering. The fabrication of 3D composite structures was performed through the synthesis of inorganic particles within a biopolymer macromolecular network during 3D printing process. The formation of a new CP phase was studied through X-ray diffraction, Fourier transform infrared spectroscopy and scanning electron microscopy. Both the phase composition and the diameter of the CP particles depend on the concentration of a liquid component (i.e., the “ink”). The 3D printed structures were fabricated and found to have large interconnected porous systems (mean diameter ≈800 μm) and were found to possess compressive strengths from 0.45 to 1.0 MPa. This new approach can be effectively applied for fabrication of biocompatible scaffolds for bone tissue engineering constructions.

## Introduction

3D printing is one promising methodology for tissue engineering constructions with specific architectonics and properties. It has the attractive advantages of both accurate and reproducible layer-by-layer fabrication of complex-shaped structures [1–4]. A number of biocompatible materials, such as polymers of different nature (both natural and synthetic), as well as a variety of calcium phosphates (CPs) are used for this purpose [2]. In this respect, the alginate-based materials are of particular interest. Alginate (extracellular polysaccharide) is a popular biomaterial because of a number of key advantages: convenient precursors, nontoxic, excellent biocompatibility and appropriate biodegradability [5–7]. Additionally, CPs are widely used for bone graft substitution due to their chemical affinity to the bone mineral content [8]. One of the strategies to improve the bioactivity of the polymer-based materials is to incorporate some inorganic phase, such as CP particles, into their structure [9–11]. The 3D printing of these materials is usually achieved by simple ink jet processing of a mechanical mixture of starting ingredients (mineral/polymer components), producing desirable structures directly through layer-by-layer manufacture of the desired product [10]. Therefore, the final 3D product consists of a mechanical mixture of the polymer slurry with more-or-less homogeneously distributed CP particles.

In this work we propose a new biomimetic approach in which 3D printing of composite structures involves a chemical interaction of the polymer slurry with a liquid “ink”, leading to in situ formation of a CP phase in the final product. It is well known that alginate allows precipitation of inorganic phases within its macromolecular network [7] and this process provides inorganic phases with different crystals sizes and morphologies [7,12]. For all these reasons, 3D printing of bioinspired structures has gained considerable interest since its inception, primarily because it can lead to artificial bone grafts that are close to native bone. In our experiments the formation of a new inorganic phase during the 3D printing process was monitored by X-ray diffraction (XRD), Fourier transform infrared (FTIR) spectroscopy and scanning electron microscopy (SEM), enabling us to find optimal parameters for the developed route.

## Results and Discussion

**Figure 1:**
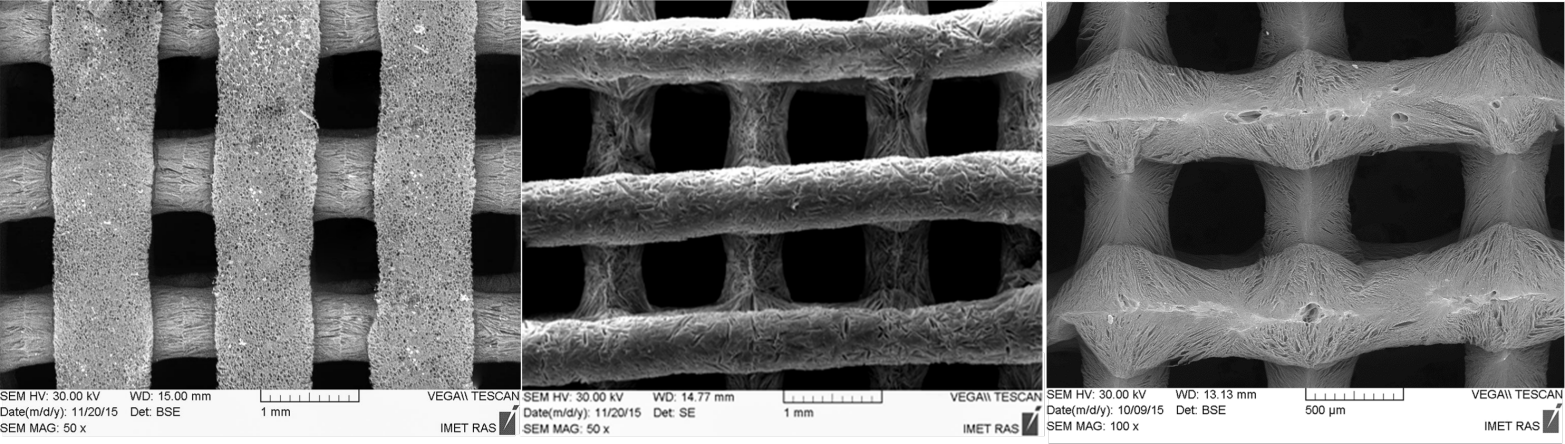
SEM micrographs of 3D printed samples.

3D printing is a powerful tool for the production of custom-designed and complex bone substitutes [2–3]. In our work, the fabrication of 3D composite structures was performed using a binary system based on aqueous solutions of sodium alginate containing PO_4_^3−^ groups. A calcium chloride aqueous solution, as a second component, was used as a source of Ca^2+^ ions. The 3D composite structures were fabricated in a cubic shape (8 × 8 × 5 mm^3^), comprising 30 interconnected longitudinal channels with dimensions of ≈800 × 800 μm^2^ running through the samples (Figure 1). This structure resulted in optimal pores sizes and good interconnectivity, which are of great importance for the design of 3D bone substitutes for biomedical applications.

The CP phase was formed upon mixing of the polymer slurry containing ammonium hydrogen phosphate with a calcium chloride aqueous solution during the printing process according to the following reaction:


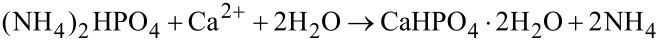


**Figure 2:**
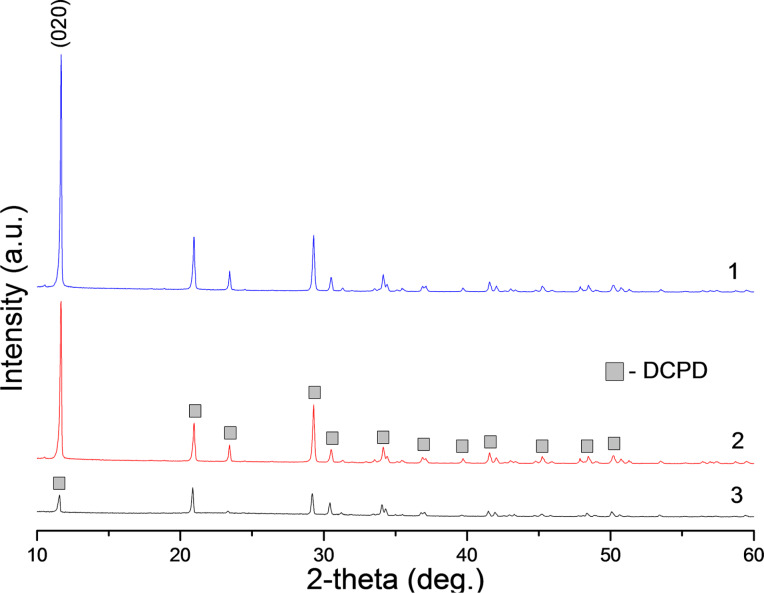
XRD spectra of 3D printed samples on the basis alginate with concentration (1) 0.25 wt %, (2) 1.0 wt % and (3) 2.0 wt %.

In fact, the mixture of the phosphate source with alginate gives rise to a homogeneous gel, suggesting an interaction between the biopolymer and the HPO_4_^2−^. The stability and homogeneity of this gel can be explained by possible interactions between the HPO_4_^2−^ moiety and the carboxylate group of alginate. This physical bonding translates to mixtures that are rich in electronic pairs leading to a higher reactivity and mineralization potential that can be transformed into composite materials [13]. According to X-ray diffraction data, synthesis in the presence of glutamic acids in the reaction medium maintained at pH 4.5 ± 0.5 yields the formation of dicalcium phosphate dihydrate (DCPD) in the printed samples (Figure 2). When precipitation is performed at a higher pH, as reported in [7,14], the difference of electrical charges in alginate is greater. As a consequence, a more compact complex without compositional water is obtained. The formation of anhydrous dicalcium phosphate (monetite) would be then kinetically favored. The estimated average mineral contents are 90, 70 and 54% of DCPD for alginate concentrations of 0.25, 1.0 and 2.0 wt %, respectively.

**Figure 3:**
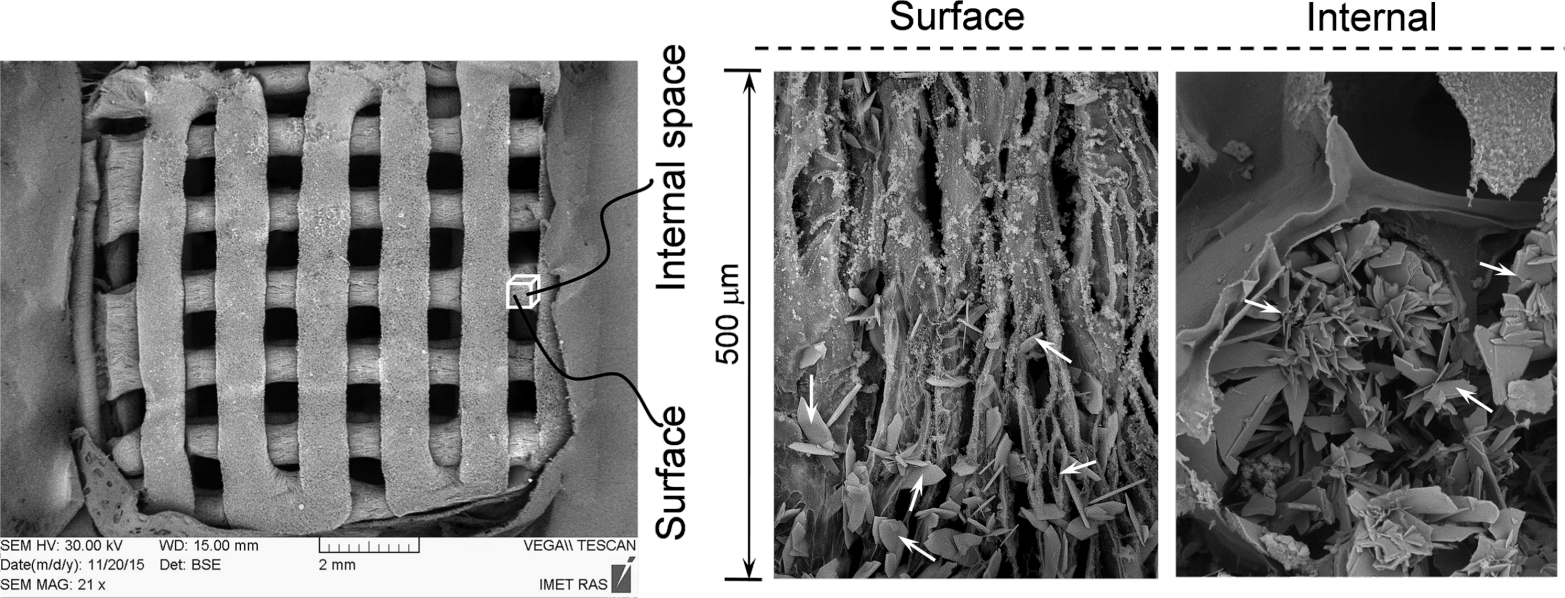
SEM images showing a general sample overview and details of the surface and internal region of a 3D printed sample (white arrows indicate DCPD crystals).

**Figure 4:**
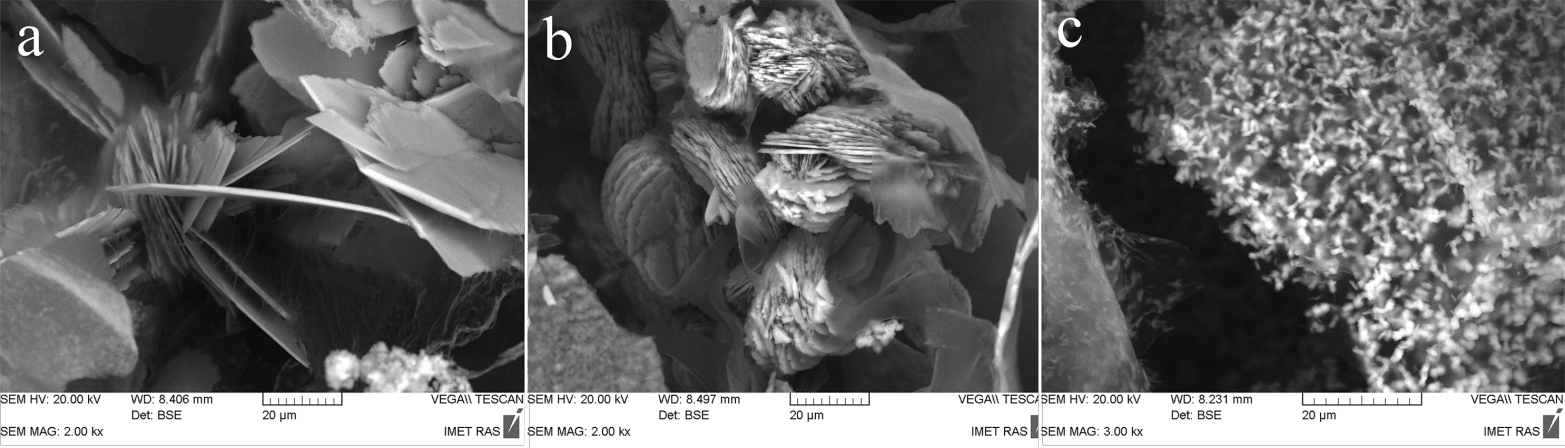
SEM micrographs of the microstructure of 3D printed samples on the basis of alginate with concentration of (a) 0.25, (b) 1.0 and (c) 2.0 wt %.

Figure 3 shows SEM images of 3D printed samples, where inorganic matrix DCPD crystal is prepared with a 1 M solution of ammonium hydrogen phosphate and alginate concentration of 0.25 wt %. The morphology of the crystals show plate- and needle-like structures with different sizes. The formation of DCPD crystals is observed on the surface and internal regions of the 3D printed samples (Figure 3). Moreover, the SEM images demonstrate a decrease of plate-shaped DCPD particle for sizes from ≈40 to 1 μm with increasing alginate concentration from 0.25 wt % up to 2.0 wt % (Figure 4). This effect may be caused by an increase in concentration of heterogeneous nucleation centers of a calcium phosphate phase on the carboxyl groups of the amino acids. X-ray diffraction data lend support to this tendency: the height of the strongest peak (020) of DCPD decreases by a factor of 7 as the alginate and, correspondently, amino acid concentration increases from 0.25 to 2.0 wt % (Figure 2).

**Figure 5:**
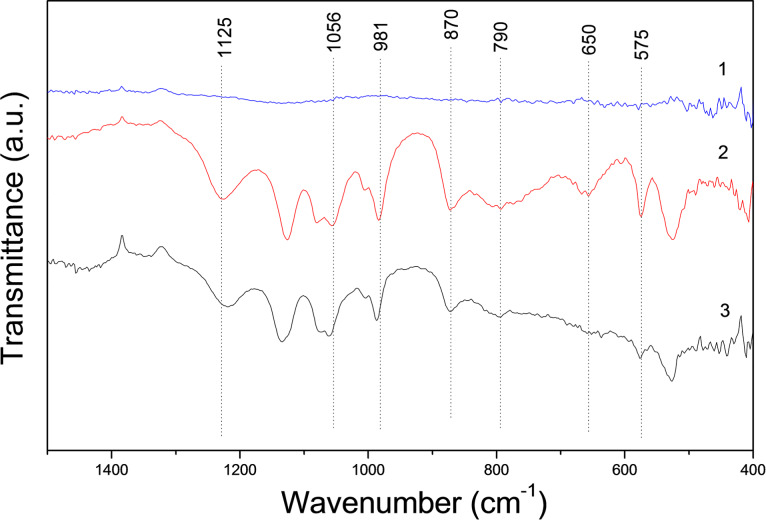
FTIR spectra of (1) sodium alginate, (2) DCPD, and (3) the 3D printed product.

The FTIR spectra of DCPD exhibits principal characteristic bands known to be associated with РО_4_ (ν_4_) at 575 and 1125 cm^−1^, (ν_1_) at 981 cm^−1^, (ν_3_) at 870 cm^−1^ and 1056 cm^−1^; as well as H_2_O at 650 cm^−1^, 790 cm^−1^ [15]. In our FTIR spectra of 3D printed DCPD–alginate samples, the bands are shifted from 1125 cm^−1^ for DCPD to 1114 cm^−1^ and from 1056 to 1064 cm^−1^. The bands at 981 cm^−1^, 870 cm^−1^, 575 cm^−1^ and 520 cm^−1^ shifted insignificantly (Figure 5). The H_2_O bands were observed to shift from 650 to 636 cm^−1^. Apparently, calcium phosphate reacts with alginate through the carboxyl groups of amino acids and calcium in calcium phosphate, because the frequency changes are observed for groups located near calcium ions in the phosphate structures [13].

**Figure 6:**
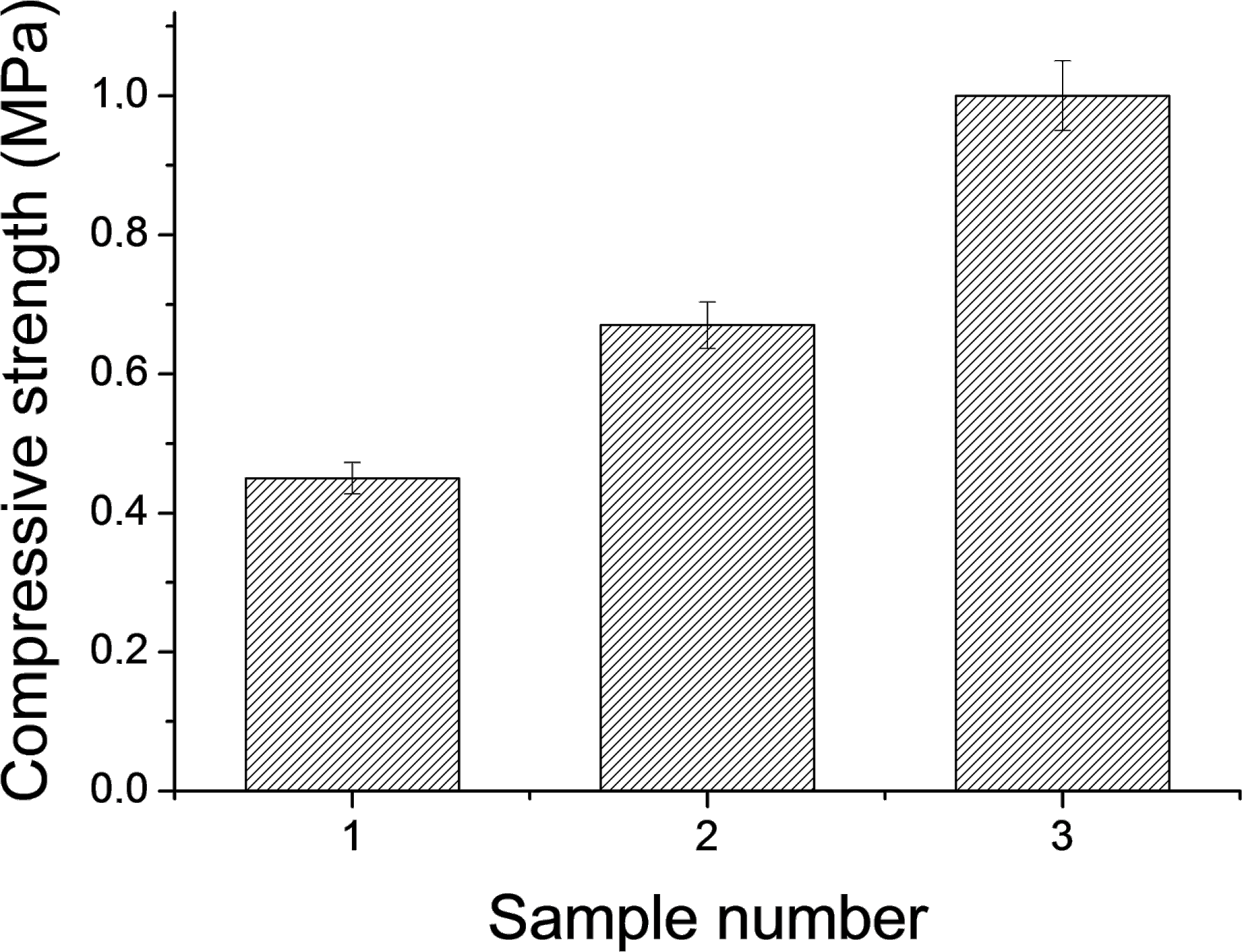
Compressive strength of 3D printed samples on the basis of alginate with concentration of (1) 0.25, (2) 1.0 and (3) 2.0 wt %.

The compressive strength of 3D printed samples is shown in Figure 6. The mechanical properties of 3D printed samples are relatively low due to the weak bonding between different printed layers. However, the compressive strength of composite materials increased with alginate concentration from 0.45 MPa up to about 1.0 MPa at *p* ≤ 0.005. The increase in the compressive strength can be explained by an increase in alginate content within the 3D printed samples. Similar to our result it has been reported in the literature [16] that in situ precipitated calcium phosphate in polymeric composites tend to have lower mechanical strength.

The chemical and phase composition of the developed 3D printed samples can be adjusted further by chemical post-treatment. For instance, the hydrolysis of DCPD might lead to the development of an octacalcium phosphate phase and an adhesive effect between particles could take place [17].

## Conclusion

We propose a new “biomimetic + 3D printing” approach for fabrication of complex-structured composite bone substitutes using appropriate raw materials and a new “ink”. Depending on the processing parameters and conditions, it is possible to achieve materials with adjustable mechanical properties, specified composition, morphology and CP crystal size. Our results may provide a new approach for synthesis of highly osteogenic composite materials and for the effective fabrication of custom-designed implants and tissue engineering constructs via 3D printing on the commercial scale.

## Experimental

### Materials

The fabrication of 3D composite structures was performed using a binary system based on aqueous solutions of sodium alginate. The polymer slurry of sodium alginate (CAS number 9005-38-3) with concentrations of 0.25, 1.0 and 2.0 wt % was prepared in distilled water in presence of glutamic acid (C_5_H_9_NO_4_) (CAS number 56-86-0) with concentrations of 0.25, 1.0 and 2.0 wt %. Glutamic acid was used to maintain a low pH value. Then, a solution of ammonium hydrogen phosphate (CAS number 7783-28-0) with concentration of 5 wt % was added as the source of phosphorus in the system. The second “ink” was calcium chloride (CAS number 10043-52-4) water solution with a concentration of 10 wt %, being the source of calcium. All reagents were purchased from Sigma-Aldrich.

### 3D printing

**Figure 7:**
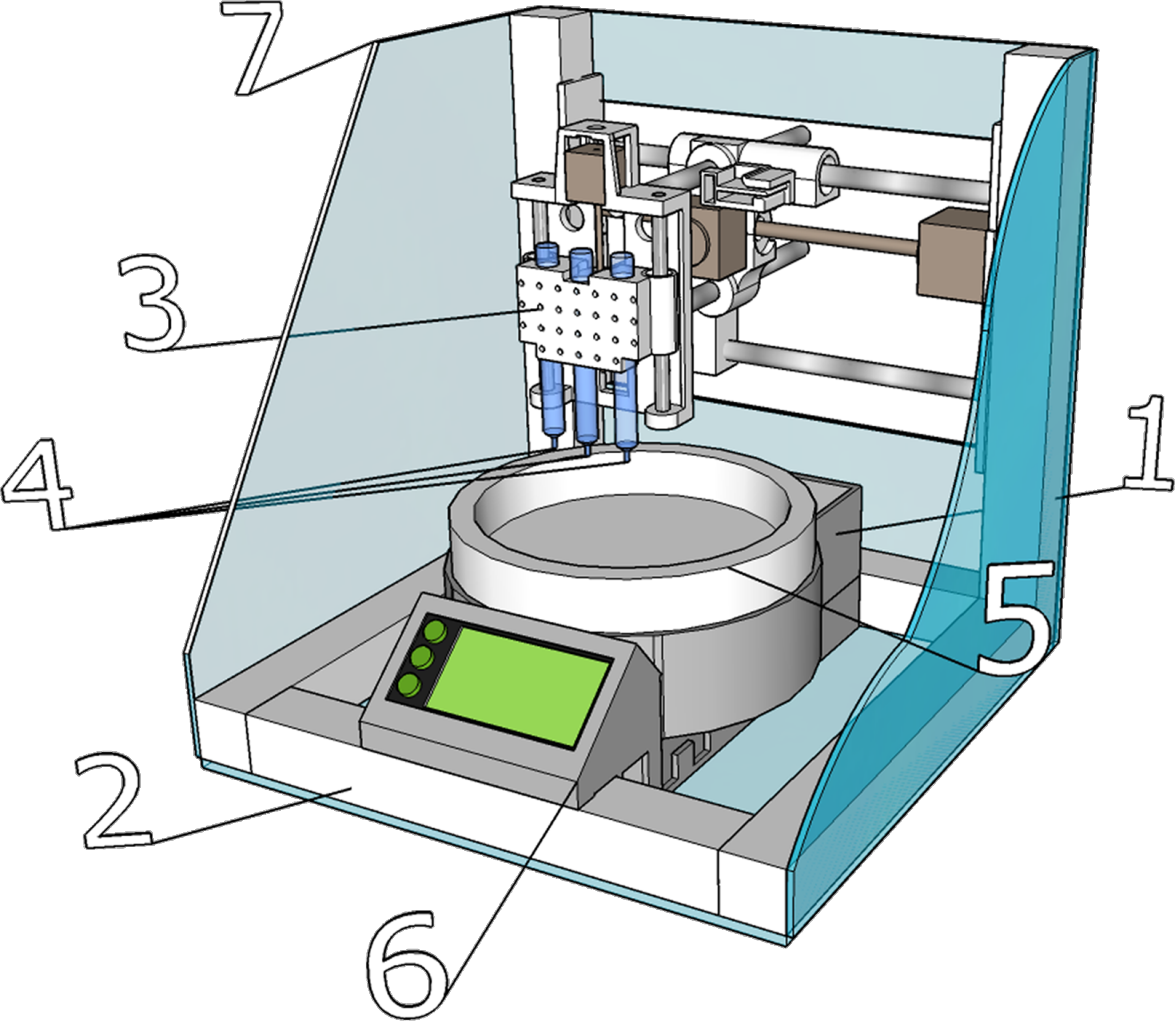
Custom-designed 3D printer. 1 - power supply; 2 - metal frame; 3 - printing head with a reservoir and heaters; 4 - nozzles; 5 - thermostatically controlled worktable; 6 - microcontroller unit and power electronic modules; 7 - sealed enclosure.

A custom-designed 3D printer was used for our experiments, as shown in Figure 7. The initial reactant solutions were placed in two separate cartridges of 3D printer. Thereafter, they were injected through a nozzle (or needle, depending on the initial viscosity of the solutions) of disposable syringe on a cooled (−5 to −30 °C) flat glass substrate. The pH of the reaction medium was measured by an Econix-Expert 001 pH meter (Econix-Expert Ltd., Moscow, Russia) and maintained at pH 4.5 ± 0.5. The printed samples were deep-frozen at −50 °C and then freeze-dried at 6 × 10^−5^ atm for 10–12 h. Finally, the fabricated 3D structure was washed in distilled water and incubated at 37 °C for one day.

### Characterization

As described in [17], the phase composition of the samples was analyzed by conventional X-ray diffraction (XRD) technique (Shimadzu XRD-6000, Japan, Ni-filtered Cu Kα_1_ target, λ = 1.54183 Å). The samples were scanned at a 2θ angle from 10–60° with a 0.02° step and a preset time of 5 s. A scanning electron microscopy apparatus (Tescan Vega II, Czech Republic), operated in secondary and backscattered electron modes, was used for 3D microstructure analysis. The samples were sputter-coated with a 25 nm thick gold layer prior to imaging, imparting electrical conductivity to the surfaces. FTIR spectroscopy (Nicolet Avatar 330, England) was performed after mixing 1 mg of the grinded sample with 300 mg of KBr powder followed by compacting into a thin pellet in a stainless steel die with a 1 cm inner diameter. FTIR data were recorded over the range of 4000–400 cm^−1^ with 128 scans.

As described in [17] the compressive strength of the samples was evaluated in accordance with the ISO standard 83.100: Cellular materials. Five samples for each point were used. Compression testing was carried out using an Instron 5581 (Bucks, UK) testing machine operating at a crosshead speed of 1 mm/min. Statistical analysis was performed using SPSS software, version 17.0 (Statistical Package for Social Sciences, SPSS Inc., USA). The mean and standard deviation of compressive strength were calculated.
